# Antioxidant and Anti-Inflammatory Effects of Thyme (*Thymus vulgaris* L.) Essential Oils Prepared at Different Plant Phenophases on *Pseudomonas aeruginosa* LPS-Activated THP-1 Macrophages

**DOI:** 10.3390/antiox11071330

**Published:** 2022-07-06

**Authors:** Edina Pandur, Giuseppe Micalizzi, Luigi Mondello, Adrienn Horváth, Katalin Sipos, Györgyi Horváth

**Affiliations:** 1 Department of Pharmaceutical Biology, Faculty of Pharmacy, University of Pécs, Rókus u. 2., H-7624 Pécs, Hungary; edina.pandur@aok.pte.hu (E.P.); horvath.adrienn2@pte.hu (A.H.); katalin.sipos@aok.pte.hu (K.S.); 2 Department of Chemical, Biological, Pharmaceutical and Environmental Sciences, University of Messina, 98168 Messina, Italy; giumicalizzi@unime.it (G.M.); lmondello@unime.it (L.M.); 3 Chromaleont s.r.l., c/o Department of Chemical, Biological, Pharmaceutical and Environmental Sciences, University of Messina, 98168 Messina, Italy; 4 Unit of Food Science and Nutrition, Department of Medicine, University Campus Bio-Medico of Rome, 00128 Rome, Italy; 5 Department of Pharmacognosy, Faculty of Pharmacy, University of Pécs, Rókus u. 2., H-7624 Pécs, Hungary

**Keywords:** thyme, essential oil, macrophage, antioxidant capacity, inflammation, cytokines

## Abstract

Thyme (*Thymus vulgaris* L.) essential oil (TEO) is widely used as an alternative therapy especially for infections of the upper respiratory tract. TEO possesses antiviral, antibacterial, and antifungal properties. The emerging antibiotic resistance of bacterial strains, including *Pseudomonas aeruginosa*, has prompted the urge to find alternative treatments. In the present study, we examined the anti-inflammatory and antioxidant effects of thymol, the main compound of TEO, and two TEOs prepared at the beginning and at the end of the flowering period that may make these oils promising candidates as complementary or alternative therapies against *P. aeruginosa* infections. The activity measurements of the antioxidant enzymes peroxidase (PX), catalase (CAT), and superoxide dismutase (SOD) as well as the determination of total antioxidant capacity of *P. aeruginosa*-activated THP-1 cells revealed that thymol and both TEOs increased CAT and SOD activity as well as the antioxidant capacity of the THP-1 cells. The measurements of the proinflammatory cytokine mRNA expression and secreted protein level of LPS-activated THP-1 cells showed that from the two TEOs, only TEO prepared at the beginning of the flowering period acted as a potent inhibitor of the synthesis of IL-6, IL-8, IL-β, and TNF-α. Our results suggest that not only thymol, but also the synergism or the antagonistic effects of the additional compounds of the essential oils are responsible for the anti-inflammatory activity of TEOs.

## 1. Introduction

Thyme (*Thymus vulgaris* L.) essential oil (TEO) is widely used as an alternative therapy for certain diseases, such as expectorant in the infections of the upper respiratory tract [1]. Due to its biological activity, TEO possesses antiviral, antibiofilm, antibacterial, and antifungal properties [2,3]. Recently, it was also revealed that TEO acts as an anticancer agent by providing an antineoplastic effect [4].

The biological activity of TEO may depend on its chemical composition. The major component of the EO is thymol, which was proven to have antibacterial, antifungal, antiviral, and antihyperglycemic effects [2,5,6]. The additional components of TEO, such as carvacrol, *p*-cymene, *γ*-terpinene, *β*-myrcene, linalool, and terpinen-4-ol, may contribute to and/or modify the antimicrobial effect of TEO. It has been revealed that the different chemotypes of TEOs trigger various anti-inflammatory responses in microglial cells [7]. The chemical composition of the EOs may depend on the phenophase of the plant and the time of oil preparation [8,9,10] that can alter its biological activity.

The emerging antibiotic resistance of bacterial strains necessitates the need to find alternative treatments with antimicrobial effects [11]. *Pseudomonas aeruginosa*, a Gram-negative bacterium, is one of the most common pathogens in the human respiratory system [12]. *P. aeruginosa* infection is very prevalent in the case of chronic respiratory tract diseases (e.g., chronic obstructive pulmonary disease and COPD) and tend to relapse or cause reinfections [13,14,15]. *P. aeruginosa* infection is difficult to treat because of its intrinsic ability to resist many types of antibiotics even if combination therapy is used [12,16]. EOs prepared from different types of medicinal plants could be promising candidates as complementary or alternative therapies against *P. aeruginosa* infections.

THP-1 human monocyte/macrophage cell lines activated by *P. aeruginosa* lipopolysaccharide (LPS) or by *P. aeruginosa* itself are a suitable model for examining the effects of new therapeutic agents on the immune response [17,18,19]. *P. aeruginosa* LPS acts by binding to the Toll-like receptors (TLRs) on the cellular surface and activates the nuclear factor-κB (NFκB) signaling pathway responsible for the regulation of the transcription of pro-inflammatory cytokines (e.g., IL-6, IL-8, or TNF-α) [20,21]. The hyperinflammatory response mediated by *P. aeruginosa* LPS could result in severe tissue damage in the case of pneumonia, cystic fibrosis, or COPD [17,22,23].

The production of reactive oxygen species (ROS) is one of the most important ways to eliminate microorganisms [24]. The long term and high level of ROS contributes to the hyperactivation of proinflammatory cytokine production of macrophages [25]. Moreover, the ROS released into the extracellular space could participate in tissue damage [26].

In the present study, we focused on the antioxidant and anti-inflammatory effects of TEOs prepared at two different phenophases: the beginning and end of the flowering period. The EOs used in the experiments were distilled from fresh thyme plants cultivated in Hungary (Szigetvár city, Baranya County).

To determine the antioxidant effect of thymol, the main compound of TEO, and the two TEOs, THP-1 cells were treated with *P. aeruginosa* LPS for different time periods. We examined both the preventative effect (pretreatment with TEOs) and the attenuating effect (TEO treatment after LPS treatment) of thymol and TEOs on ROS production and on the activity of antioxidant enzymes (peroxidase, superoxide dismutase, and catalase). To prove the effectiveness of thymol and TEOs on reducing proinflammatory cytokine transcription and synthesis, we examined IL-6, IL-β, IL-8, and TNF-α mRNA and protein levels of differently treated THP-1 cells compared to ACHP (2-Amino-6-[2-(cyclopropylmethoxy)-6-hydroxyphenyl]-4-(4-piperidinyl)-3-pyridinecarbonitrile), an NFκB inhibitor [27,28] used as positive control.

Our findings suggest that the composition of the EOs modify the antioxidant as well as anti-inflammatory effects. TEO prepared at the beginning of the flowering period acted as a potent inhibitor of proinflammatory cytokine synthesis.

## 2. Materials and Methods

### 2.1. Plant Material and Distillation of Essential Oils

The plant (*Thymus vulgaris* L.) collection was carried out at the beginning of the flowering period (23 May 2019) and at the end of the flowering period (12 June 2019) in the two phenophases. The harvesting took place at Szigetvár city (Baranya county, Hungary, coordinates: (46°02′60.00″ N, 17°47′59.99″ E). TEO was obtained from the freshly collected plant material by hydrodistillation according to the *Hungarian Pharmacopoeia* 8th edition (2003). For one distillation, 100 g of dried drug part and 1000 mL of distilled water were used. The duration of the procedure was 3 h. Distillation procedures were prepared in parallel [9]. The TEO content was measured with a volumetric method: 8450 μL and 3920 µL were extracted and isolated from fresh plant material at the two phenophases, respectively. The chemical composition of the TEO samples was determined by a single-quadrupole mass spectrometer (GC-MS) and flame ionisation detector (GC-FID) [9].

### 2.2. GC-MS and GC-FID

TEO samples (10 µL) were solubilized in 990 μL of *n*-heptane (dil. 1:100) (Merck Life Science Kft., Budapest, Hungary) and were injected on the GC-MS and GC-FID systems in order to provide the complete identification and quantification of the analytes. Briefly, the separation and identification of terpene and terpenoid compounds were carried out by using a GCMS-QP2020 instrument (Shimadzu, Duisburg, Germany) equipped with a split–splitless injector (280 °C) and an AOC-20i auto-sampler. A nonpolar capillary column, namely SLB-5 ms 30 m × 0.25 mm ID × 0.25 μm d*_f_* (Merck Life Science), was used for the separation of analytes. Quantitative analyses were performed with a GC-2010 instrument (Shimadzu) equipped with a split–splitless injector (280 °C), an FID detector, and an AOC-20i auto-sampler. Chromatographic conditions included the following parameters: volume injection: 0.5 μL in split mode (1:10) and temperature program: 50 °C–300 °C at 3.0 °C/min. The helium was used as carrier gas at the linear velocity of 30 cm/s. MS parameters were as follows: mass range 40–550 amu; ion source temperature: 220 °C; and interface temperature: 250 °C. The FID parameters included detector temperature settled at 300 °C (sampling rate 40 ms), and gas flows were 40 mL/min for hydrogen, 30 mL/ min for make up (nitrogen), and 400 mL/min for air, respectively. The GCMSsolution software (version 4.50 Shimadzu) was used for data collection and processing. The FFNSC mass spectral library (version 4.0, Shimadzu) was used for the compound identification. Within such a context, two different identification parameters, namely MS spectral similarity and linear retention index (LRI) correspondence, were utilized according to our previous publication [29]. A C_7_–C_40_ Saturated Alkanes Standard mixture, 1000 μg/mL each component in hexane, (Merck Life Science) was used for the calculation of the LRIs. GC-FID analyses were acquired and processed using the LabSolution software (version 5.92, Shimadzu). Each sample was analyzed for three sequential runs for increasing the precision of data [29].

### 2.3. Cell Culture

THP-1 human monocyte/macrophage cell line was purchased from the European Collection Authenticated Cell Cultures (Merck Life Science Kft., Budapest, Hungary). The cells were maintained in RPMI-1640 medium supplemented with 10% fetal bovine serum (FBS; EuroClone S.p.A., Pero, Italy) and 1% Penicillin/Streptomycin (P/S; Lonza Ltd., Basel, Switzerland) in a humidified incubator containing 5% CO_2_ at 37 °C. THP-1 cells were placed into 96-well or 6-well plates according to the experiments and were cultured for 24 h before the treatments. The inflammation was induced by treatments using 100 ng/mL *Pseudomonas aeruginosa* LPS (*Pseudomonas aeruginosa* 10; Merck Life Science Kft., Budapest, Hungary) according to our previous publication [9]. As positive control for the inhibition of the inflammation, 5 μM of ACHP (2-Amino-6-[2-(cyclopropylmethoxy)-6-hydroxyphenyl]-4-(4-piperidinyl)-3-pyridinecarbonitrile; Tocris Bioscience, Bio-Techne R&D Systems Kft., Budapest, Hungary), an NFκB inhibitor, was used. For the antioxidant activity measurements, the THP-1 cells were treated with 1000 ng/mL *Pseudomonas aeruginosa* LPS. Thymol standard was achieved by adding 6 mg thymol to 1 mL DMSO (6 mg/mL). The cells were treated with 500-fold diluted thymol standard (Merck Life Science Kft., Budapest, Hungary) and TEOs to determine their effects on the antioxidant enzymes and on proinflammatory cytokine production. We established three experimental settings: (1) The effect of thymol standard and TEOs on proinflammatory cytokine production was determined on the THP-1 cell in the absence of LPS. (2) The anti-inflammatory effects of TEOs and thymol were determined using LPS pretreatment followed by administration of EO, (3) The protective effect of EOs and thymol was examined by using EO pretreatment then LPS treatment. Dimethyl sulfoxide (DMSO) used for preparing EO emulsions was considered as control of the treated cells. The final concentration of DMSO in the culture medium did not exceed 0.01%.

### 2.4. Cell Viability Measurements

Thymol standard and TEO stock solutions were prepared by using 100 μL of DMSO (100%, Merck Life Science Kft., Budapest, Hungary) to 900 μL of thymol and TEOs. The emulsions were mixed by rigorous vortexing, and then serial dilutions were prepared in phosphate-buffered saline (PBS, Lonza Ltd., Basel, Switzerland). We prepared 500-fold, 1000-fold, 2000-fold, and 3000-fold dilutions to determine the toxic concentration for the cells. Stock solutions and the dilutions were always prepared freshly right before each experiment. For controls, a 10% DMSO/PBS solution was diluted the same way as the EOs. The THP-1 cells were seeded into 96-well plates using 5 × 10^3^ cells/well, and they were treated with either thymol or the two different TEOs for 6 h and 24 h. Cell viability was determined using Cell Counting Kit-8 cell viability assay (Merck Life Science Kft., Budapest, Hungary) after following the treatments, according to the protocol. The plates were incubated for 1 h at 37 °C and 5% CO_2_. The optical density of the samples was measured at 450 nm using MultiSkan GO microplate spectrophotometer (Thermo Fisher Scientific Inc., Waltham, MA, USA). Viability of the treated cells was expressed as percentile compared to the nontreated cells [9].

### 2.5. Reactive Oxygen Species (ROS) Measurements

The THP-1 cells were seeded into 96-well plates using 5 × 10^3^ cells/well. The proper LPS concentration and incubation time were determined by time and concentration dependence experiments (Figure S1). After 24 h resting period, first the cells were pretreated with 1000 ng/mL LPS for 6 h and 24 h, and then they were treated using 500-fold diluted thymol or TEOs for 24 h. In the second experiment, the cells were pretreated with 500-fold diluted thymol or TEOs for 24 h followed by LPS treatments for 6 h and 24 h. To measure the ROS generating effect of LPS alone, the cells were treated with LPS for 6 h or 24 h then were treated with 500-fold diluted DMSO for 24 h. In the case of EO pretreatment, to determine the LPS-generated ROS production, the cells were treated with only 500-fold diluted DMSO for 24 h followed by LPS treatments for 6 h or 24 h. For absolute control, the cells were treated with DMSO and/or distilled water and the solvent of LPS in the same order and for the same time as in the case of the EO and LPS pretreatments. Intracellular ROS production was determined by using Fluorometric Intracellular ROS Kit (Merck Life Science Kft., Budapest, Hungary) according to the manufacturer’s protocol. Fluorescence was measured by using EnSpire Multimode Plate Reader (PerkinElmer Inc., Waltham, MA, USA).

### 2.6. Determination of Peroxidase (PX) Activity

The THP-1 cells were placed onto Petri dishes (6 cm) using 1 × 10^6^ cells/Petri dish. The cells were treated the same way as described in the ROS measurements section. PX activity was determined by Peroxidase Activity assay Kit (Merck Life Science Kft., Budapest, Hungary) according to the protocol of the manufacturer. Briefly, the cells were collected after the treatments and were homogenized with 200 μL of assay buffer. The lysates were centrifuged for 10 min at 15,000× *g*, and 50 μL of the supernatants were used for the measurements. The plates were incubated for 2 h, and the colorimetric assay was used for the determination of peroxidase activity. The absorbance was measured at 570 nm using MultiSkan GO microplate spectrophotometer (Thermo Fisher Scientific Inc., Waltham, MA, USA). The enzyme activity was expressed as mU/mL.

### 2.7. Determination of Catalase (CAT) Activity

The THP-1 cells were seeded into Petri dishes (6 cm) using 1 × 10^6^ cells/Petri dish. The cells were treated the same way as described in the ROS measurements section. Catalase activity was determined using Catalase Assay kit (Merck Life Science Kft., Budapest, Hungary) according to the manufacturer’s protocol. The cells were collected by centrifugation using 2000× *g* for 15 min at 4 °C. The cell pellets were resuspended in 1 mL of ice-cold homogenization buffer (PBS, pH 7.4, and protease inhibitor) and were sonicated for 2 × 3 min. The lysates were centrifuged at 10,000× *g* for 15 min at 4 °C. The supernatants were used for the measurements. The absorbance was measured at 540 nm using MultiSkan GO microplate spectrophotometer (Thermo Fisher Scientific Inc., Waltham, MA, USA). CAT activity was expressed as nmol/min/mL.

### 2.8. Determination of Superoxide Dismutase (SOD) Activity

The THP-1 cells were placed onto Petri dishes (6 cm) using 1 × 10^6^ cells/Petri dish. The cells were treated the same way as described in the ROS measurements section. SOD activity was measured with Superoxide Dismutase (SOD) Activity Assay Kit (Merck Life Science Kft., Budapest, Hungary). The cells were harvested by centrifugation at 2000× *g* for 5 min, and then the pellets were lysed in 200 μL of ice-cold lysis buffer (0.1 M Tris-HCl, pH 7.4; 0.5% Triton X-100, 5 mM mercaptoethanol and protease inhibitor). The lysates were clarified by centrifugation at 14,000× *g* for 5 min at 4 °C. A total of 20 μL of each supernatant was used for the measurement. The absorbance was measured at 450 nm using MultiSkan GO microplate spectrophotometer (Thermo Fisher Scientific Inc., Waltham, MA, USA). SOD activity was expressed as U/mL.

### 2.9. Determination of Total Antioxidant Capacity (TAC)

The THP-1 cells were placed onto 6-well plates using 5 × 10^5^ cells/well. The cells were treated the same way as described in the ROS measurements section. TAC was determined using Antioxidant Assay Kit (Merck Life Science Kft., Budapest, Hungary). The cells were collected by centrifugation at 2000× *g* for 5 min. The cell lysates were prepared by sonicating the cells (2 × 2 min) in 100 μL of ice-cold PBS, pH 7.4. The lysates were centrifuged at 14,000× *g* for 10 min at 4 °C. A total of 20 μL of each supernatant was used for the measurement. The absorbance was measured at 570 nm using MultiSkan GO microplate spectrophotometer (Thermo Fisher Scientific Inc., Waltham, MA, USA). The TAC was expressed as μM.

### 2.10. Real-Time PCR Analysis

THP-1 cells were seeded into 6-well plates using 5 × 10^5^ cells/well. After 24 h resting period, the cells were treated with 500-fold diluted thymol or TEOs as described earlier. After the incubation, THP-1 cells were pelleted by centrifugation at 2000× *g* for 5 min at RT. Total RNA was isolated from each sample using Aurum Total RNA Isolation Kit (Bio-Rad Inc., Hercules, CA, USA). The cDNA was synthesized from 200 ng of total RNA using iScript Select cDNA Synthesis Kit (Bio-Rad Inc., Hercules, CA, USA) in a 20 µL of total reaction volume according to the protocol of the manufacturer. The expression level of the target genes was determined using a CFX96 Real-time System (Bio-Rad Inc., Hercules, CA, USA) and with iTaq™ Universal SYBR^®^ Green Supermix (Bio-Rad Inc., Hercules, CA, USA). Relative expression rate was evaluated by the Livak (2^−∆∆Ct^) method using the Bio-Rad CFX Maestro 1.1. software (Bio-Rad Inc., Hercules, CA, USA). For normalization, the relative expression of β-actin housekeeping gene was determined. The expression level of the examined genes in DMSO-treated cells was regarded as 1. The relative mRNA expression level of the EO-treated THP-1 cells was compared to the control and was expressed as fold change [9]. The sequences of the real-time PCR primers are described in Table 1.

### 2.11. Enzyme-Linked Immunosorbent Assay (ELISA) Measurements

THP-1 cells were placed into 6-well plates using 5 × 10^5^ cells/well. After 24 h resting period, the cells were treated with 500-fold diluted thymol or TEOs as described earlier. After the incubation period, THP-1 cells were pelleted by centrifugation at 2000× *g* for 5 min at RT, and the supernatants were transferred into new tubes for ELISA measurements. The samples were stored at −80 °C until processing. The concentrations of secreted proinflammatory cytokines IL-6, IL-1β, IL-8, and TNF-α were determined from the supernatants in triplicate in each independent experiment. The measurements were carried out using human IL-6-, IL-1β-, IL-8-, and TNF-α-specific ELISA kits (Thermo Fisher Scientific Inc., Waltham, MA, USA) according to the instructions of the manufacturer.

### 2.12. Statistical Analysis

The cell viability assay and the ROS measurements were carried out in quadruplicate in each independent experiment. The peroxidase, CAT, SOD activity measurements, and TAC determinations were carried out in triplicate in three independent experiments. The real-time PCR analyses and ELISA measurements were carried out in triplicate in each independent experiment. The number of the independent experiments was indicated with *n*. Statistical analysis was performed using SPSS software (IBM Corporation, Armonk, NY, USA). Statistical significance was determined by two-way ANOVA followed by Scheffe’s post hoc test. Data are shown as mean  ±  standard deviation (SD). Statistical significance was set at *p* value < 0.05.

## 3. Results

### 3.1. Effects of Thymol and TEOs on Cell Viability of THP-1 Cells

**Figure 1:**
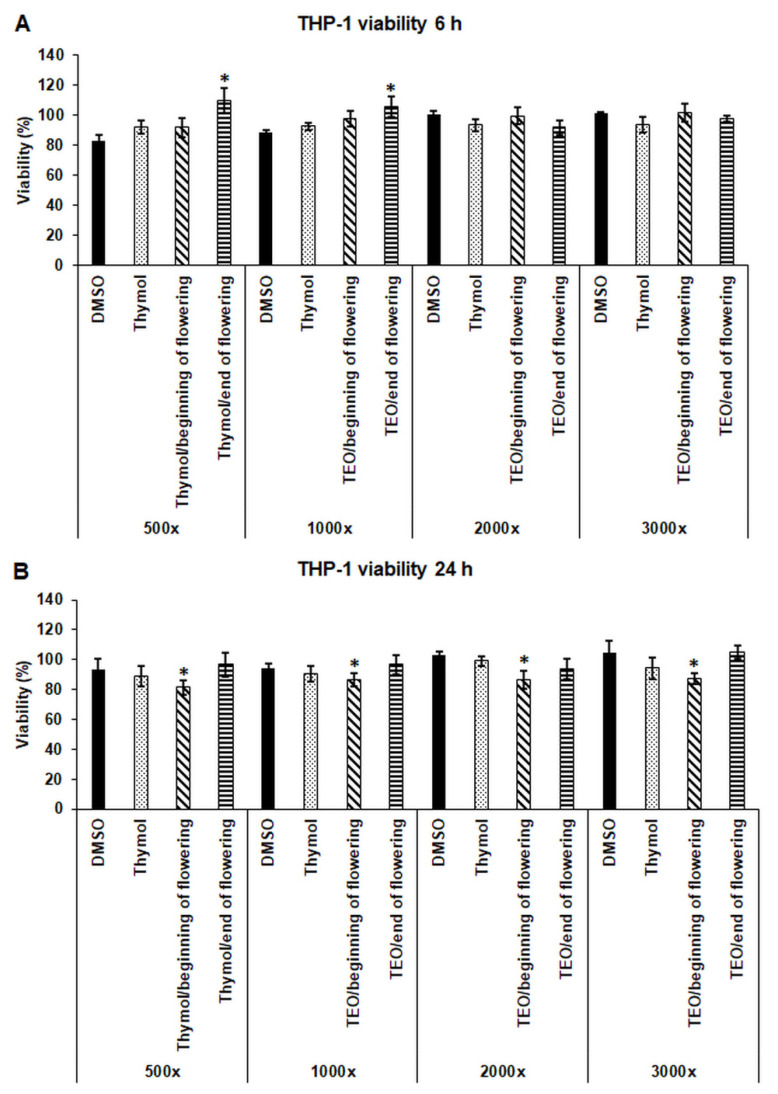
Determination of the viability of THP-1 cells treated with thymol and TEOs. Viability of the THP-1 cells was measured using CCK-8 cell viability assay after 6 h (**A**) and 24 h (**B**) treatments using serial dilutions of the stock solutions of DMSO, thymol, and TEOs. Viability is expressed as percentile of the untreated cells. The bars represent mean values, and error bars represent standard deviation (SD) for four independent experiments (n = 4). Cell viability assays were carried out in quadruplicate in each experiment. Asterisks indicate *p* < 0.05 compared to the DMSO-treated cells. Statistical analysis was carried out by two-way ANOVA followed by Scheffe’s post hoc test.

The cytotoxic effects of thymol and TEOs were determined after serial dilutions of the thymol standard and the EOs prepared at the beginning and at the end of the flowering period. No significant decrease in living cell number was found after 6 h treatments using four dilutions of the EOs (500-fold, 1000-fold, 2000-fold, and 3000-fold) compared to the DMSO control (Figure 1A). After 24 h, a significant alteration of cell viability was measured only in the case of treatment using TEO/beginning of flowering, but there was no difference between the effect of the different dilutions (Figure 1B). Based on the results, the 500-fold dilution of thymol and TEOs was chosen for the further experiments.

### 3.2. Composition of the Essential Oils Prepared at the Beginning and at the End of Flowering Period

Seventy-one compounds were identified in TEO/beginning of the flowering period, and seventy-two compounds were determined in TEO/end of the flowering period (Table 2). Two compounds, (*Z*)-jasmone and *α*-amorphene, were present only in TEO/end of the flowering period, and one compound, thymol acetate, was identified only in TEO/beginning of the flowering period. The major components of the TEOs were thymol (55.81% and 54.21%), *p*-cymene (12.89% and 20.64%), *γ*-terpinene (15.18% and 6.01%), carvacrol (2.3% and 2.9%), linalool (1.46% and 2.15%), (*E*)-caryophyllene (1.56% and 1.92%), myrcene (1.45% and 1.28%), *α*-terpinene (1.4% and 0.82%), and *α*-thujene (0.99% and 0.99%) (Table 2). The different composition of the two TEOs may contribute to the alteration on cell viability after treatment with TEO/beginning of flowering.

### 3.3. Effects of Thymol and TEOs on Reactive Oxygen Species (ROS) Generated by LPS

**Figure 2:**
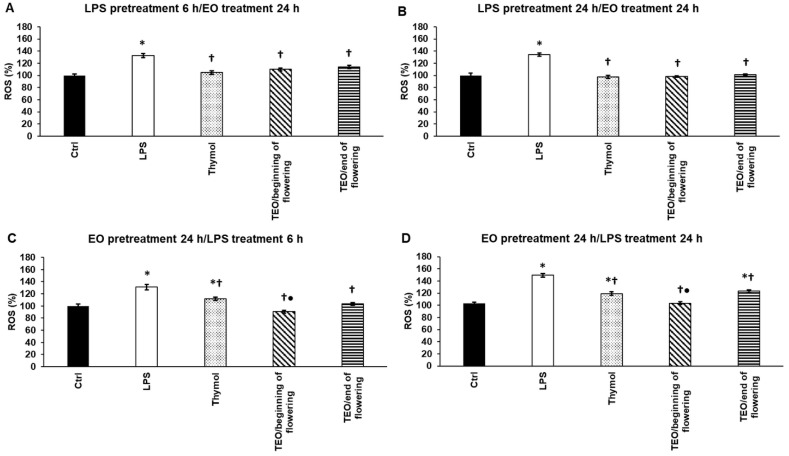
Determination of the effects of thymol and TEOs on reactive oxygen species (ROS) generated by LPS. As a control, the THP-1 cells were treated with DMSO, the carrier of EOs or distilled water, and the solvent of LPS in the same order and for the same time as in the case of the EO and LPS pretreatments. For LPS treatment, the cells were treated with LPS for 6 h or 24 h, and then they were treated with 500-fold diluted DMSO for 24 h or were treated with DMSO for 24 h, and then they were treated with LPS for 6 h and 24 h. For LPS pretreatment, the cells were treated with LPS for 6 h and 24 h, and then they were treated using 500-fold diluted thymol or TEOs for 24 h. For EO pretreatment, the cells were treated with 500-fold diluted thymol or TEOs for 24 h followed by LPS treatments for 6 h and 24 h. Intracellular ROS production was determined by using Fluorometric Intracellular ROS Kit and was expressed as % compared to the control. The bars represent mean values, and error bars represent standard deviation (SD) for three independent experiments (n = 3). The assays were carried out in quadruplicate in each experiment. Asterisk indicates *p* < 0.05 compared to the control. Cross marks *p* < 0.05 compared to LPS treatment. Bullet marks *p* < 0.05 compared to TEO/end of flowering. Statistical analysis was carried out by two-way ANOVA followed by Scheffe’s post hoc test.

To examine whether thymol or TEOs possess antioxidant capacity, THP-1 cells were treated with *P. aeruginosa* LPS to trigger ROS production. The effects of TEOs were tested before and after LPS administration to determine if TEOs reduce the level of ROS or prevent ROS production. Thymol and TEOs significantly decreased ROS after 6 h and 24 h LPS treatments suggesting that they have antioxidant capacity (Figure 2A,B). Using thymol and TEO pretreatments, all three samples were able to decrease ROS compared to LPS treatments, but TEO/beginning of flowering was significantly more effective than thymol or TEO/end of flowering (Figure 2C,D).

### 3.4. Effects of Thymol and TEOs on Peroxidase (PX) Activity of LPS-Treated THP-1 Cells

**Figure 3:**
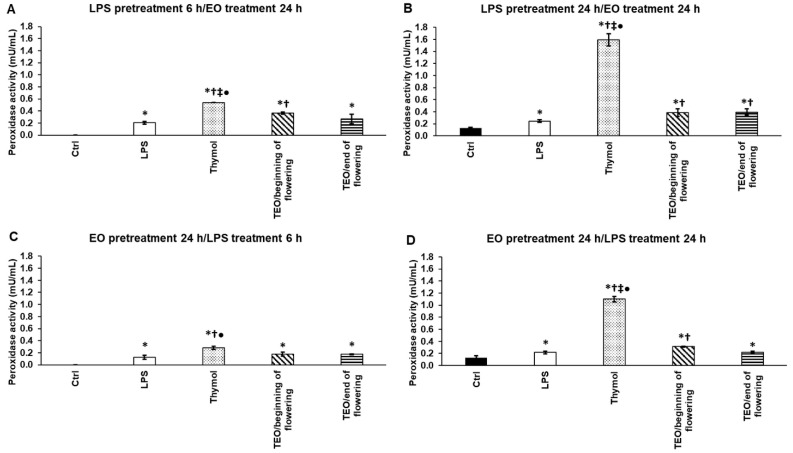
Determination of the effects of thymol and TEOs on peroxidase activity (PX) of the THP-1 cells. As a control, the THP-1 cells were treated with DMSO, the carrier of EOs or distilled water, and the solvent of LPS in the same order and for the same time as in the case of the EO and LPS pretreatments. For LPS treatment, the cells were treated with LPS for 6 h or 24 h, and then 500-fold diluted DMSO was added for 24 h, or the cells were treated with DMSO for 24 h, and then LPS was administered for 6 h and 24 h. For LPS pretreatment, the cells were incubated with LPS for 6 h and 24 h, and then 500-fold diluted thymol or TEOs were added for 24 h. For EO pretreatment, the cells were treated with 500-fold diluted thymol or TEOs for 24 h followed by LPS addition for 6 h and 24 h. Peroxidase activity was determined by Peroxidase Activity Assay Kit according to the protocol of the manufacturer and was expressed as mU/mL. The bars represent mean values, and error bars represent standard deviation (SD) for three independent experiments (n = 3). The assays were carried out in triplicate in each experiment. Asterisk indicates *p* < 0.05 compared to control. Cross marks *p* < 0.05 compared to LPS treatment. Double cross shows *p* < 0.05 compared to TEO/beginning of flowering. Bullet marks *p* < 0.05 compared to TEO/end of flowering. Statistical analysis was carried out by two-way ANOVA followed by Scheffe’s post hoc test.

We examined the effects of thymol and TEOs on the activities of antioxidant enzymes: peroxidase, catalase, and superoxide dismutase. LPS administration increased peroxidase activity (Figure 3A–D). Thymol significantly elevated PX activity after 6 h and 24 h LPS pretreatments, and the level of activity was higher in the case of the longer LPS treatment (Figure 3A,B). After 6 h LPS pretreatment, only TEO/beginning of flowering was able to increase PX activity (Figure 3A), but it was less effective compared to thymol. In the case of 24 h LPS pretreatment, both TEOs significantly increased PX activity compared to LPS addition, but their effect was significantly lower compared to thymol (Figure 3B). In the case of EO pretreatments, thymol significantly increased PX activity even using 6 h or 24 h LPS treatments after adding EOs (Figure 3C,D). Between the two TEOs, only TEO/beginning of flowering could elevate the activity of PX (Figure 3D).

### 3.5. Effects of Thymol and TEOs on Catalase (CAT) Activity of LPS-Treated THP-1 Cells

**Figure 4:**
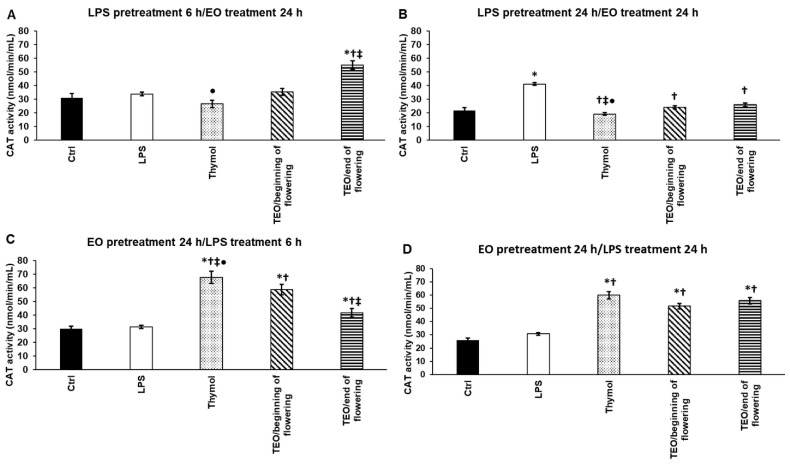
Determination of the effects of thymol and TEOs on catalase (CAT) activity of the THP-1 cells. As a control, the THP-1 cells were treated with DMSO, the carrier of EOs or distilled water, and the solvent of LPS in the same order and for the same time as in the case of the EO and LPS pretreatments. For LPS treatment, the cells were treated with LPS for 6 h or 24 h, and then they were treated with 500-fold diluted DMSO for 24 h, or they were treated with DMSO for 24 h, and then they were treated with LPS for 6 h and 24 h. For LPS pretreatment, the cells were treated with LPS for 6 h and 24 h, and then they were treated using 500-fold diluted thymol or TEOs for 24 h. For EO pretreatment, the cells were treated with 500-fold diluted thymol or TEOs for 24 h followed by LPS treatments for 6 h and 24 h. CAT activity was determined using Catalase Assay kit according to the protocol of the manufacturer and was expressed as nmol/min/mL. The bars represent mean values, and error bars represent standard deviation (SD) for three independent experiments (n = 3). The assays were carried out in triplicate in each experiment. Asterisk indicates *p* < 0.05 compared to control. Cross marks *p* < 0.05 compared to LPS treatment. Double cross shows *p* < 0.05 compared to TEO/beginning of flowering. Bullet marks *p* < 0.05 compared to TEO/end of flowering. Statistical analysis was carried out by two-way ANOVA followed by Scheffe’s post hoc test.

We also measured the activity of catalase, the hydrogen peroxide-degrading enzyme after LPS and EO pretreatments. Interestingly, *P. aeruginosa* LPS was not able to increase CAT activity only after 24 h pretreatment (Figure 4B). After 6 h LPS pretreatment, only TEO/end of flowering elevated CAT activity (Figure 4A). The 24 h EO administrations significantly decreased catalase activity after the longer LPS pretreatments instead of increasing it (Figure 4B). The effect of thymol was the highest; it significantly elevated CAT activity compared to TEOs. Although both TEOs increased enzyme activity, TEO/beginning of flowering was more effective when 6 h LPS was added to the cells (Figure 4C). In the case of EO pretreatment followed by 24 h LPS treatment, all three samples significantly increased CAT activity compared to LPS treatment (Figure 4D). These results show that TEOs and thymol provide a preventative effect against oxidative damage.

### 3.6. Effects of Thymol and TEOs on Superoxide Dismutase (SOD) Activity of LPS-Treated THP-1 Cells

**Figure 5:**
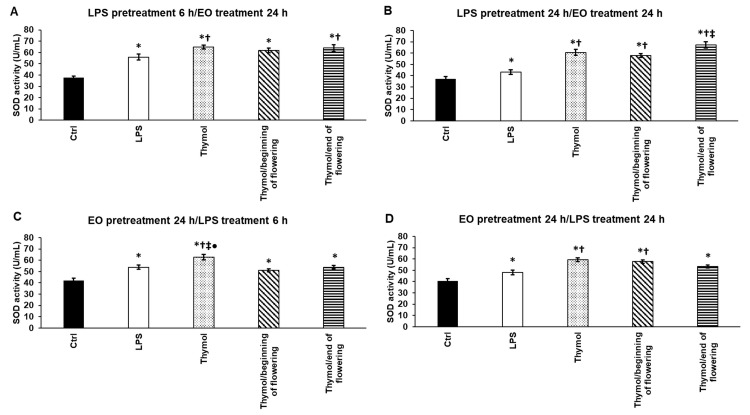
Determination of the effects of thymol and TEOs on superoxide dismutase (SOD) activity of the THP-1 cells. As a control, the THP-1 cells were treated with DMSO, the carrier of EOs or distilled water, and the solvent of LPS in the same order and for the same time as in the case of the EO and LPS pretreatments. For LPS treatment, the cells were treated with LPS for 6 h or 24 h, and then they were treated with 500-fold diluted DMSO for 24 h or were treated with DMSO for 24 h, and then LPS was added for 6 h and 24 h. For LPS pretreatment, the cells were treated with LPS for 6 h and 24 h, and then they were treated using 500-fold diluted thymol or TEOs for 24 h. For EO pretreatment, the cells were treated with 500-fold diluted thymol or TEOs for 24 h followed by LPS administration for 6 h and 24 h. SOD activity was measured with Superoxide Dismutase (SOD) Activity Assay Kit according to the protocol of the manufacturer and was expressed as U/mL. The bars represent mean values, and error bars represent standard deviation (SD) for three independent experiments (n = 3). The assays were carried out in triplicate in each experiment. Asterisk indicates *p* < 0.05 compared to control. Cross marks *p* < 0.05 compared to LPS treatment. Double cross shows *p* < 0.05 compared to TEO/beginning of flowering. Bullet marks *p* < 0.05 compared to TEO/end of flowering. Statistical analysis was carried out by two-way ANOVA followed by Scheffe’s post hoc test.

The superoxide radical-eliminating enzyme (SOD) activity was also examined after LPS and EO pretreatments. LPS significantly elevated SOD activity in each treatment type (Figure 5A–D). Thymol was also successful in significantly increasing SOD activity in each treatment compared to the effect of LPS on THP-1 cells (Figure 5A–D). TEO/end of flowering caused a significant increase in SOD activity after 6 h LPS pretreatment compared to the LPS administration alone (Figure 5A). TEO/beginning of flowering acted similarly to LPS treatment alone (Figure 5A). After the 24 h LPS pretreatment, TEO/end of flowering was more effective compared to both LPS and TEO/beginning of flowering administrations (Figure 5B), although TEO/beginning of flowering was also capable of significantly increasing SOD activity compared to the LPS addition (Figure 5B). In the case of EO pretreatments, thymol generated a significantly stronger effect on SOD than TEOs (Figure 5C). In the case of EO pretreatment followed by the addition of LPS for 24 h, both TEOs activated SOD, but only TEO/beginning of flowering caused a significant alteration compared to LPS treatment (Figure 5D).

### 3.7. Effects of Thymol and TEOs on Total Antioxidant Capacity (TAC) of LPS-Treated THP-1 Cells

**Figure 6:**
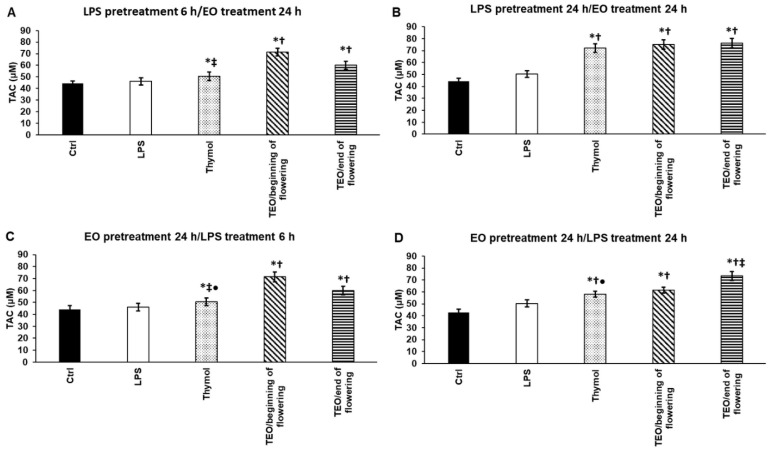
Determination of the effects of thymol and TEOs on total antioxidant capacity (TAC) of the THP-1 cells. As a control, the THP-1 cells were treated with DMSO, the carrier of EOs or distilled water, and the solvent of LPS in the same order and for the same time as in the case of the EO and LPS pretreatments. For LPS treatment, the cells were treated with LPS for 6 h or 24 h, and then they were treated with 500-fold diluted DMSO for 24 h, or they were treated with DMSO for 24 h, and then LPS was added for 6 h and 24 h. For LPS pretreatment, the cells were treated with LPS for 6 h and 24 h, and then they were treated using 500-fold diluted thymol or TEOs for 24 h. For EO pretreatment, the cells were treated with 500-fold diluted thymol or TEOs for 24 h followed by LPS treatments for 6 h and 24 h. TAC was determined using Antioxidant Assay Kit according to the protocol of the manufacturer and was expressed as μM. The bars represent mean values, and error bars represent standard deviation (SD) for three independent experiments (n = 3). The assays were carried out in triplicate in each experiment. Asterisk indicates *p* < 0.05 compared to control. Cross marks *p* < 0.05 compared to LPS treatment. Double cross shows *p* < 0.05 compared to TEO/beginning of flowering. Bullet marks *p* < 0.05 compared to TEO/end of flowering. Statistical analysis was carried out by two-way ANOVA followed by Scheffe’s post hoc test.

The total antioxidant capacity of the differently treated THP-1 cells was measured including the small molecule antioxidants (e.g., vitamins and glutathione) to determine which EO possesses the highest antioxidant capacity. *P. aeruginosa* LPS did not cause significant elevation of TAC (Figure 6A–D). After 6 h LPS pretreatment, all of the examined TEOs and thymol increased TAC, but TEO/beginning of flowering treatment resulted in the highest concentration of TAC (Figure 6A). The THP-1 cells showed higher TAC after 24 h LPS pretreatment/EO treatments (Figure 6B). In the case of EO pretreatments, both TEOs were more effective compared to LPS and thymol administrations (Figure 6C,D). It was revealed that TEO/beginning of flowering acted more efficiently to elevate TAC in the case of EO pretreatment/6 h LPS addition (Figure 6C). Meanwhile, the opposite result was found in the case of EO pretreatment/24 h LPS treatment: TEO/end of flowering was more effective (Figure 6D). According to the results, it seems that TEO/beginning of flowering provides the largest TAC (Figure 6A–D).

### 3.8. Effects of Thymol and TEOs on mRNA Expression and Secretion of Proinflammatory Cytokines IL-6, IL-8, IL-1β, and TNF-α

First, we determined the effects of thymol and TEOs on the mRNA levels of the pro-inflammatory cytokines, IL-6, IL-8, IL-1β, and TNF-α in THP-1 cells.

**Figure 7:**
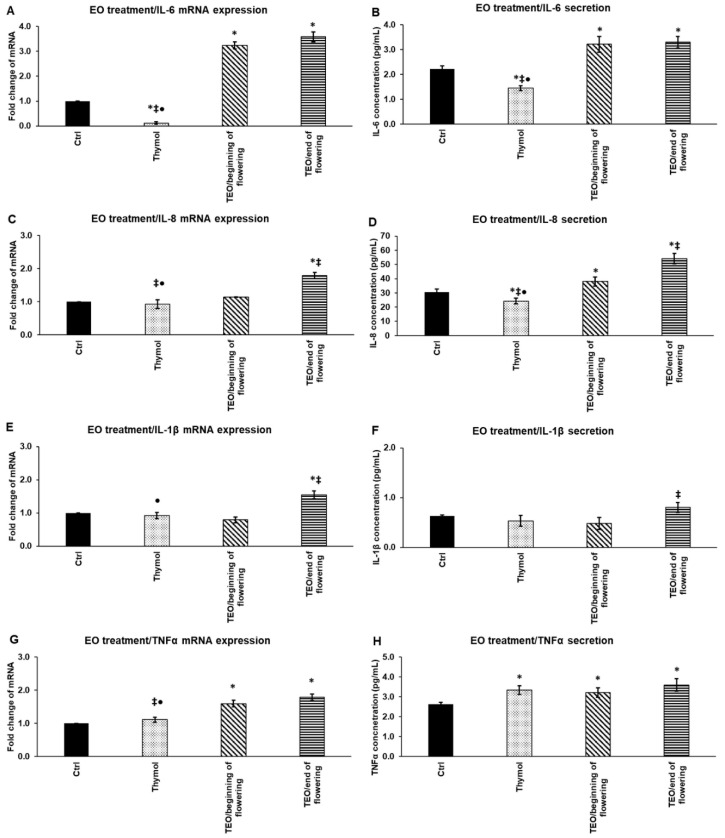
Determination of mRNA expression (**A**,**C**,**E**,**G**) and protein (**B**,**D**,**F**,**H**) levels of proinflammatory cytokines IL-6, IL-8, IL-1β, and TNF-α after thymol and TEO treatments of THP-1 cells. THP-1 cells were treated with 500-fold diluted thymol and TEOs for 24 h. DMSO-treated cells were used as a control of the EO-treated cells. Real-time PCR for the proinflammatory cytokines was performed with SYBR green protocol. Β-actin was used as housekeeping gene, and the relative expression of controls was regarded as 1. Proinflammatory cytokine secretions were determined using IL-6-, IL-8-, IL-1β-, and TNF-α-specific ELISA kits according to the manufacturer’s protocols. The bars represent mean values, and error bars represent standard deviation (SD) for three independent determinations (n = 3). Real-time PCR and ELISA measurements were carried out in triplicate in each independent experiment. Asterisks indicate *p* < 0.05 compared to control. Double cross shows *p* < 0.05 compared to TEO/beginning of flowering. Bullet marks *p* < 0.05 compared to TEO/end of flowering. Statistical analysis was carried out by two-way ANOVA followed by Scheffe’s post hoc test.

Thymol did not significantly change the mRNA expression of proinflammatory cytokines except IL-6, which decreased after 24 h thymol treatment (Figure 7A). TEO/beginning of flowering did not alter IL-8 and IL-1β mRNA expression (Figure 7C,E), but significantly increased IL-6 and TNF-α mRNA levels compared to the control (Figure 7A,G). TEO/ end of flowering elevated the mRNA levels of all four examined cytokines (Figure 7A,C,E,G). Moreover, TEO/ end of flowering significantly increased IL-8 and IL-1β mRNA expression compared to thymol and TEO/beginning of flowering (Figure 7C,E).

The secreted cytokine levels were also quantified to reveal any differences or delays between mRNA and protein levels. The amount of the secreted proteins changed parallel with the mRNA expression levels of proinflammatory cytokines. Thymol decreased the protein levels of the cytokines, except TNF-α, which was significantly elevated compared to the control (Figure 7B,D,F,H). TEO/beginning of flowering significantly increased IL-6 and IL-8 levels compared to both the control and thymol and elevated TNF-α secretion compared to the control (Figure 7B,D,H). TEO/end of flowering raised the protein levels of all four cytokines, but with different rates. The IL-6, IL-8, and TNF-α secretion was significantly higher compared to the control (Figure 7B,D,H). Meanwhile, the IL-8 and IL-1β secretion mediated by TEO/end of flowering was found to be significantly higher compared to TEO/beginning of flowering (Figure 7D,F).

These results show that treatments with TEOs alone act as an activator of the monocytes, since both increased the expression and production of the examined proinflammatory cytokines. On the other hand, the main component of TEOs, thymol, mainly decreased the proinflammatory cytokine expression suggesting that the composition of TEOs may have an impact on the transcription and synthesis of cytokines.

### 3.9. Inhibitory Effect of Thymol, TEOs, and ACHP NFκB Inhibitor on mRNA Expression and Secretion of Proinflammatory Cytokines after P. aeruginosa LPS Pretreatment

We examined the effects of thymol and TEOs on the proinflammatory cytokine mRNA and protein expressions after the treatment of the THP-1 cells with *P. aeruginosa* LPS for 24 h. To determine the degree of the effectiveness of the examined EOs, an NFκB inhibitor ACHP was used as positive control.

**Figure 8:**
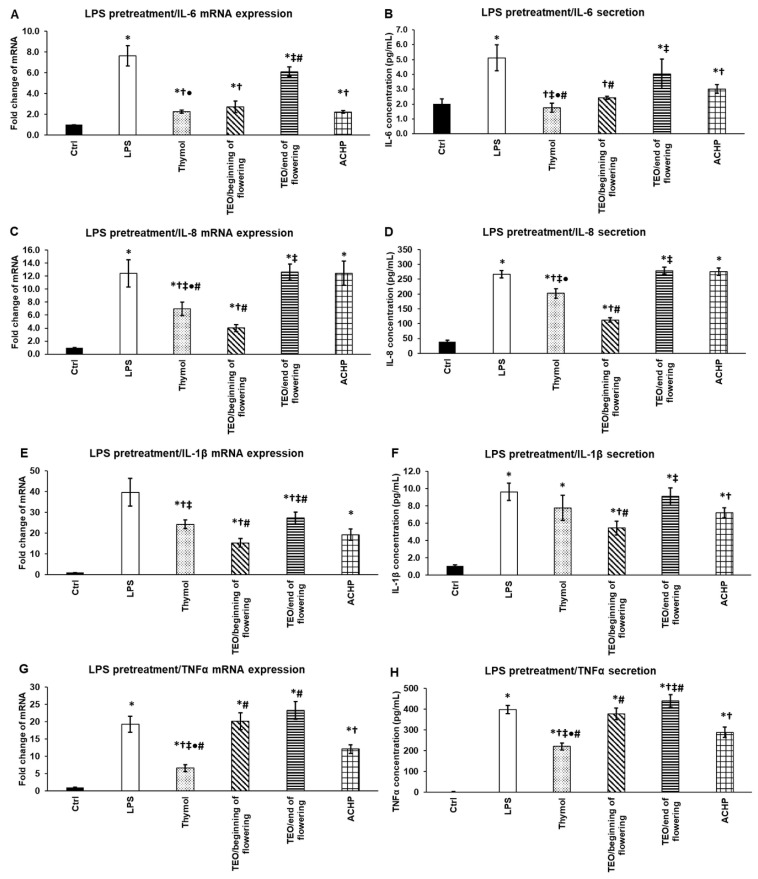
Effects of thymol, TEOs, and ACHP NFκB inhibitor on mRNA (**A**,**C**,**E**,**G**) and protein (**B**,**D**,**F**,**H**) levels of proinflammatory cytokines IL-6, IL-8, IL-1β, and TNF-α after LPS pretreatment. THP-1 cells were pretreated with 100 ng/mL LPS for 24 h and then with 500-fold diluted thymol, TEOs, or 5 μM ACHP for 24 h. DMSO-treated cells were used as control of the treated cells. Real-time PCR for the proinflammatory cytokines was performed with SYBR green protocol. β-actin was used as housekeeping gene, and the relative expression of controls was regarded as 1. Pro-inflammatory cytokine secretions were determined using IL-6-, IL-8-, IL-1β-, and TNF-α-specific ELISA kits according to the manufacturer’s protocols. The columns represent mean values, and error bars represent standard deviation (SD) for three independent experiments (n = 3). The assays were carried out in triplicate in each experiment. Asterisk indicates *p* < 0.05 compared to control. Cross marks *p* < 0.05 compared to LPS treatment. Double cross shows *p* < 0.05 compared to TEO/beginning of flowering. Bullet marks *p* < 0.05 compared to TEO/end of flowering. Number sign indicates *p* < 0.05 compared to ACHP treatment. Statistical analysis was carried out by two-way ANOVA followed by Scheffe’s post hoc test.

Thymol and TEO/beginning of flowering were able to significantly decrease IL-6 mRNA expression compared to LPS treatment, and their effect was almost as efficient as ACHP (Figure 8A). At the protein level, thymol and TEO/beginning of flowering were significantly more efficient in reducing IL-6 secretion compared to LPS as well as the ACHP NFκB inhibitor (Figure 8B), suggesting that both thymol and TEO/beginning of flowering inhibit the NFκB signaling pathway.

In the case of IL-8, both at the mRNA and protein levels, thymol and TEO/beginning of flowering significantly reduced IL-8 levels compared to LPS and ACHP (Figure 8C,D). Moreover, TEO/beginning of flowering was more effective than thymol. 

Thymol and both TEOs as well as ACHP decreased IL-1β mRNA expression compared to LPS treatment, but only TEO/beginning of flowering was more powerful than the ACHP NFκB inhibitor (Figure 8E). At the protein level, only TEO/beginning of flowering significantly reduced IL-1β secretion compared to LPS as well as ACHP (Figure 8F). These observations suggest that in the case of the two EOs, there is a delay between the mRNA expression and protein secretion, which may be due to the post-translational modification of IL-1β.

The examination of TNF-α mRNA and protein levels revealed that only thymol could reduce its expression, but its effect was stronger compared to the ACHP NFκB inhibitor (Figure 8G,H).

Based on these results, we assume that TEO/beginning of flowering is a powerful inhibitor of IL-6, IL-8, and IL-1β mRNA and protein syntheses, but is ineffective inthe inhibition of TNF-α. The effect of TEO/beginning of flowering is comparable to ACHP, suggesting that this EO influences the activity of the NFκB pathway. Since thymol was almost as effective as TEO/beginning of flowering, and thymol was the only one that was capable of acting on TNF-α production, we suppose that not only the thymol component but the additional compounds of TEO contribute to the inhibitory effect of the EOs.

### 3.10. Pretreatments with Thymol, TEOs, and ACHP NFκB Inhibitor Prevent the mRNA Expression and Secretion of Proinflammatory Cytokines of THP-1 Cells Exposed to P. aeruginosa LPS

The preventive effect of thymol and TEOs on inflammation was also examined. EO pretreatments were used for 24 h, which were followed by a 24 h LPS treatment. The effectiveness of thymol and TEOs was compared to that of ACHP.

**Figure 9:**
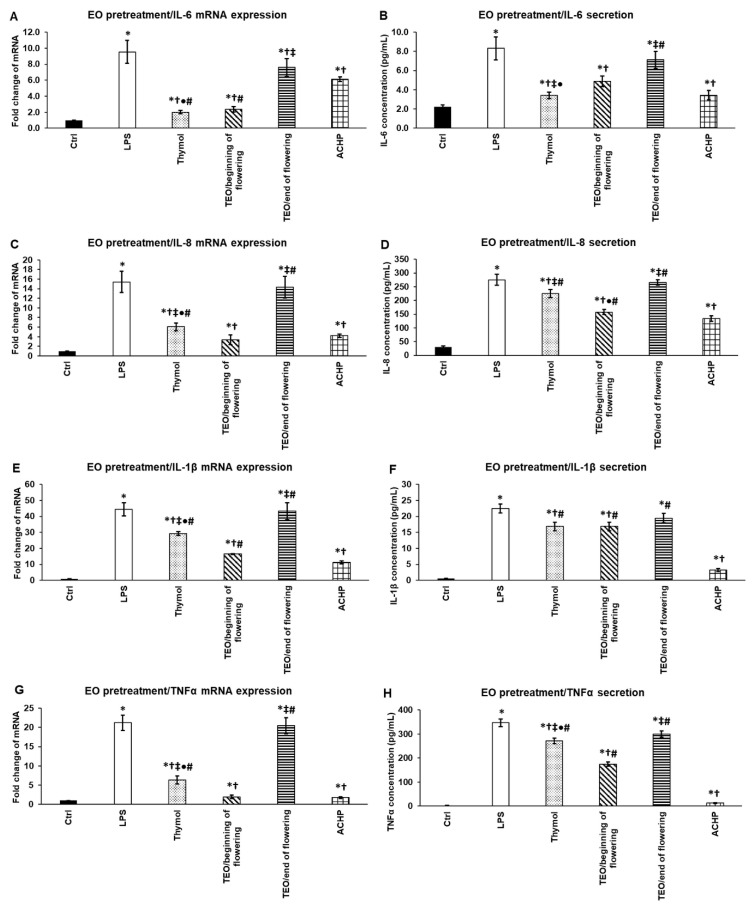
Effects of thymol, TEOs, and ACHP NFκB inhibitor pretreatments on mRNA (**A**,**C**,**E**,**G**) and protein (**B**,**D**,**F**,**H**) levels of proinflammatory cytokines IL-6, IL-8, IL-1β, and TNF-α. THP-1 cells were pretreated with 500-fold diluted thymol, TEOs, and 5 μM ACHP for 24 h and then with 100 ng/mL LPS for 24 h. DMSO administration was used as a control of the treated cells. Real-time PCR for the proinflammatory cytokines was performed with SYBR green protocol. β-actin was used as housekeeping gene, and the relative expression of controls was regarded as 1. Proinflammatory cytokine secretions were determined using IL-6-, IL-8-, IL-1β-, and TNF-α-specific ELISA kits according to the manufacturer’s protocols. The columns represent mean values, and error bars represent standard deviation (SD) for three independent experiments (n = 3). The assays were carried out in triplicate in each experiment. Asterisk indicates *p* < 0.05 compared to control. Cross marks *p* < 0.05 compared to LPS treatment. Double cross shows *p* < 0.05 compared to TEO/beginning of flowering. Bullet marks *p* < 0.05 compared to TEO/end of flowering. Number sign indicates *p* < 0.05 compared to ACHP treatment. Statistical analysis was carried out by two-way ANOVA followed by Scheffe’s post hoc test.

In the case of IL-6, both thymol and TEO/beginning of flowering significantly reduced the IL-6 mRNA level compared to LPS, and they were significantly more efficient than the ACHP NFκB inhibitor (Figure 9A). At the protein level, thymol was as efficacious as ACHP, and TEO/beginning of flowering was also capable of significantly reducing IL-6 secretion compared to LPS treatment (Figure 9B).

TEO/beginning of flowering showed the highest activity in attenuating IL-8 mRNA as well as IL-8 protein levels, although ACHP was more efficient in decreasing IL-8 pro-inflammatory cytokine expression (Figure 9C,D). The main component of TEO, thymol, also significantly downregulated IL-8 synthesis compared to LPS (Figure 9C,D).

Thymol and TEO/beginning of flowering were able to reduce the IL-1β mRNA level, but ACHP administration seemed to be more effective compared to LPS treatment (Figure 9E). At the protein level, both thymol and TEO/beginning of flowering treatments resulted in the same IL-1β protein level, which was still lower compared to LPS treatment (Figure 9F). Despite this, none of the examined EOs reached the same effect as the ACHP NFκB inhibitor (Figure 9F).

In the case of TNF-α pro-inflammatory cytokine expression, TEO/beginning of flowering was the most powerful in decreasing its level followed by thymol, but neither of them was more auspicious than ACHP (Figure 9G,H).

Interestingly, TEO/end of flowering treatments were ineffective in decreasing the four examined proinflammatory cytokine expression both at the mRNA and protein levels (Figure 9A–H), suggesting that the differences in TEO compositions could act on and modify their anti-inflammatory effects.

## 4. Discussion

The emerging number of multidrug-resistant bacterial strains requires the development and utilization of alternative or complementary therapies, which are suitable for confining the infections or decreasing the probability of reinfections [30]. EOs from various medicinal plants have been used as alternative medicines in many diseases (respiratory infections, intestinal infections, skin diseases as a topical agent, etc.) [31,32].

The main compound of TEO is thymol that possesses antibacterial, antiviral, antifungal, and anti-inflammatory properties [2,5,6]. Additional major components of TEO, such as carvacrol, *p*-cymene, *γ*-terpinene, and *α*-terpinene, also have antimicrobial, antioxidant, and anticancer activities [33,34,35,36].

Based on our knowledge about the effects of TEO [2,3], we prepared EOs from the flowers of thyme plants cultivated in Hungary at two plant phenophases: at the beginning of flowering and at the end of flowering. Since thyme flowers are usually collected at the main blooming period for EO production, the examination of the anti-inflammatory and antioxidant properties of TEOs distilled at the beginning and at the end of flowering may give insight into whether the phenophases influence their activities and if they are efficient agents against inflammation. The composition of the TEOs was determined using GC-MS analysis. No significant difference was revealed in the thymol content of the TEOs (55.81% and 54.21%), but we found remarkable differences in *p*-cymene (12.89% and 20.64%) and *γ*-terpinene (15.18% and 6.01%). According to these data, the collection time of the thyme flowers has a deep impact on the chemical composition.

The antioxidant and anti-inflammatory effects of thymol and the two TEOs were examined using THP-1 human monocyte/macrophage cells activated by *P. aeruginosa* LPS.

LPS from the bacterial cell wall binds to the TLRs on the plasma membrane of macrophages [37]. Upon LPS binding, the TLR activates the downstream signaling pathways, such as NFκB, MAPK, and IRF3. The NFκB transcription factors are translocated into the nucleus and activate the transcription of the inflammatory genes, such as IL-6, IL-1β, IL-8, and TNF-α [38]. Reducing the synthesis of the proinflammatory cytokines by macrophages is a crucial point in relieving inflammation and protecting the respiratory system.

LPS of *P. aeruginosa* induces the production of ROS causing the overproduction of proinflammatory cytokines of macrophages, contributing to tissue injury [25,26]. In the case of both short-term or long-term LPS treatments, the main TEO compound, thymol, as well as the two TEOs attenuated the intracellular ROS production either by direct scavenging or by increasing the antioxidant capacity of the THP-1 macrophages. When monitoring the preventative function of TEOs, it seems that TEO/beginning of flowering was the most effective compared to thymol and TEO/end of flowering.

To see whether EOs trigger the action of antioxidant enzymes, the activities of peroxidase (PX), catalase (CAT), and superoxide dismutase (SOD) were measured. Thymol significantly increased the activity of PX and SOD but reduced CAT activity after LPS treatment. Meanwhile, it elevated the activity of all three enzymes in the case of thymol pretreatment. According to the literature, it seems that the antioxidant effect of thymol depends on the applied concentration, the cell type (Caco-2 colon carcinoma, V79 hamster fibroblast, neutrophils, macrophages, etc.), and the utilized inducer of oxidative stress (e.g., hydrogen peroxide, menadione, LPS, etc.) [39,40,41]. In our experiments, TEO/beginning of flowering significantly elevated the activity of PX and SOD but reduced CAT activity after LPS treatment. Meanwhile, it raised the activity of all three enzymes in the case of TEO pretreatment. The action of TEO/ beginning of flowering was similar to that of thymol. TEO/end of flowering was less efficient in altering PX activity, but it was the most powerful in increasing CAT activity in the case of LPS pretreatment. On the other hand, TEO/end of flowering acted at the same rate or less in the case of EO pretreatment followed by LPS treatment compared to thymol or TEO/beginning of flowering considering CAT and SOD activities. The possible reason for the differences in the effectiveness of the EOs compared to thymol may be the concentration of the main compound, which is 1182 ng/mL in thymol, 1071.55 ng/mL in TEO/beginning of flowering, and 1040.83 ng/mL in TEO/end of flowering. The discrepancy between the effects of the two TEOs on the antioxidant enzymes suggests that the concentration differences of the constituents and/or the synergism or inhibitory effect of the compounds modify the mechanism of action. The p-cymene (20.64%), linalool (2.15%), carvacrol (2.9%), and (*E*)-caryophyllene (1.92%) were detected at higher levels in TEO/end of flowering. These compounds also possess antioxidant activity [40,42,43,44]. TEO/beginning of flowering contained more myrcene (1.45%)-, *α*-terpinene (1.4%)-, and *γ*-terpinene (15.18%)-active compounds, which also have antioxidant effects [45,46,47]. The higher levels of the aforementioned components may be the reason for the stronger effect of TEO/beginning of flowering in the case of EO pretreatment.

The intracellular total antioxidant capacity (TAC) measurements suggest that TEOs act actively as ROS scavenging agents and modify the activity of the antioxidant enzymes [41]. THP-1 cells treated with TEO/beginning of flowering were found to have the highest TAC after short-term LPS treatment, suggesting that it has the highest ROS scavenging activity. In the case of long-term LPS pretreatment all three EOs acted at the same level, suggesting both scavenging- and enzyme-triggering functions. TEO/end of flowering pretreatment followed by long-term LPS treatment raises the possibility that it enhances the effects not only of enzymatic but also of nonenzymatic antioxidants, such as glutathione [48].

Alveolar macrophages, members of the innate immune system, begin to synthetize and release chemokines, such as IL-8, and proinflammatory cytokines, such as IL-1β, IL-6, and TNF-α, given a *P. aeruginosa* infection [49]. The proinflammatory molecules contribute to the elimination of the bacterial cells, but their overproduction leads to tissue injury of the respiratory system [26]. To reveal the anti-inflammatory properties of TEOs, the proinflammatory cytokine expression of the treated THP-1 cells was determined. Only thymol was able to decrease IL-6 and IL-8 at both the mRNA and protein levels. TEO/beginning of flowering increased the IL-6, IL-8, and TNF-α levels. These findings suggest that the EOs in the absence of inflammatory molecules are able to regulate the NFκB signaling pathway, but it seems that they generate different responses. The secreted cytokines may act in an autocrine way on the THP-1 cells and trigger the further expression of IL-6, IL-8, or TNF-α [50,51].

The major components of TEOs may also contribute to their effects on macrophages. Thymol is known to possess anti-inflammatory effects by reducing IL-6, IL-1β, and TNF-α transcription via the downregulation of the NFκB pathway [52]. Carvacrol-inhibited TNF-α and IL-1β expression by modulating the c-Jun N-terminal kinase (JNK), signal transducer and activator of transcription (STAT3), activator protein-1 (AP-1), and nuclear factors of activated T-cells (NFATs) transcription factors [53]. The *p*-cymene and myrcene suppressed the LPS-induced TNF-α and IL-6 production by decreasing the activity of NFκB and mitogen-activated protein kinase (MAPK) in RAW 264.7 macrophages [54,55]. *α*-Terpinene and *γ*-terpinene as well as terpinene-4-ol can suppress the release of inflammatory mediators [56,57,58]. Linalool also possesses an anti-inflammatory effect by decreasing the phosphorylation rate of the NFκB transcription factors and reducing the production of IL-6 and TNF-α [7].

Our findings indicate that thymol and TEO/beginning of flowering were effective in decreasing IL-6, IL-1β, and IL-8 mRNA as well as protein levels of LPS-activated THP-1 macrophages. Moreover, TEO/beginning of flowering was more efficient compared to thymol in the case of IL-1β and IL-8. In spite of this, both thymol and TEO/beginning of flowering significantly decreased IL-6 secretion compared to ACHP. Interestingly, in the case of TNF-α, only thymol could successfully decrease its level. These results underlie the importance of the composition of the EOs and suggest that the components may strengthen or reduce the anti-inflammatory effect maybe via the modulation of the activity of the intracellular signaling proteins. Since ACHP blocks the NFκB and STAT3 signaling pathways [28] and was not as potent as thymol and TEO/beginning of flowering, additional regulatory mechanisms could operate in the control of cytokine production, such as C/EBPβ [59]. 

An examination of the preventive function of thymol and TEOs on proinflammatory cytokine expression revealed that TEO/beginning of flowering possessed the highest potential to reduce the mRNA levels of IL-6, IL-1β, IL-8, and TNF-α. These results suggest that the active compounds of TEO may inhibit or reduce the activation of the NFκB signaling pathway. Interestingly, in the case of IL-1β, the decreasing level of mRNA was not followed by a reduction of the secreted protein level. A possible reason for this observation is that *P. aeruginosa* LPS induces the activation of an inflammasome that maintains IL-1β secretion [60,61], although the NFκB pathway is inhibited by the EO. TEO/end of flowering did not show any anti-inflammatory properties strengthening the role of the interactions among the different compounds.

Based on our results, we have proven that both TEOs increase the antioxidant capacity of the THP-1 cells, but only TEO/beginning of flowering is a suitable inhibitor of the synthesis of IL-6, IL-8, IL-β, and TNF-α of THP-1 cells. Our results also support the relevance of the utilization of TEO that is produced from thyme flowers collected before the full blooming period as antioxidant and anti-inflammatory treatments.

## 5. Conclusions

It has been revealed that TEO distilled at the beginning of the flowering period may act as a promising regulator of ROS elimination and an inhibitor of IL-6, IL-8, IL-β, and TNF-α synthesis of THP-1 cells making it an effective and potential alternative therapy for respiratory diseases in the future.

## Figures and Tables

**Table 1:** Real-time PCR gene primers.

Primer	Gene Accession Number	Sequence 5′ → 3′
IL-6 forward	NM_000600.5	CTGAGAAAGGAGACATGTAACAAG
IL-6 reverse		GGCAAGTCTCCTCATTGAATC
IL-8 forward	NM_000584.4	CAGTGCATAAAGACATACTCC
IL-8 reverse		CACTCTCAATCACTCTCAGT
IL-1β forward	NM_000576.3	GAAATGATGGCTTATTACAGTGG
IL-1β reverse		GGTGGTCGGAGATTCGTA
TNFα forward	NM_000594.4	CTCTCTCTAATCAGCCCTCT
TNFα reverse		CTTGAGGGTTTGCTACAACA
β-actin forward	NM_007393.5	AGAAAATCTGGCACCACACC
β-actin reverse		GGGGTGTTGAAGGTGTCAAA

**Table 2:** Percentage (%) and relative concentrations (ng/mL) of the compounds of TEOs prepared at the beginning and at the end of the flowering period.

				Percentage of Compound in the Thyme Essential Oils ^a^		Relative Concentration of Compound in the Experiments (ng/mL) ^b^	
Compounds	MS Sim	LRI Exp	LRI Ref	Beginning of Flowering	End of Flowering	Beginning of Flowering	End of Flowering
Tricyclene	94	922	923	0.02	0.02	0.34	0.34
*α*-Thujene	98	925	927	0.99	0.99	18.51	18.51
*α*-Pinene	97	933	933	0.61	0.69	10.72	12.13
Camphene	97	949	953	0.39	0.50	7.02	9.00
Sabinene	95	972	972	0.02	0.01	0.36	0.18
Pent-4-enyl propanoate	94	974	974	0.03	0.01	0.50	0.17
*β*-Pinene	92	977	978	0.20	0.21	3.49	3.62
Vinyl amyl carbinol	95	979	978	0.27	0.42	4.52	7.03
Octan-3-one	93	984	986	0.03	0.03	0.48	0.48
Myrcene	96	988	991	1.45	1.28	23.20	20.48
Octan-3-ol	96	997	999	0.04	0.03	0.65	0.49
*α*-Phellandrene	96	1006	1007	0.15	0.10	2.53	1.69
*δ*-3-Carene	96	1009	1009	0.08	0.08	1.38	1.38
*α*-Terpinene	98	1017	1018	1.40	0.82	22.40	13.12
*p*-Cymene	96	1025	1025	12.89	20.64	221.71	355.01
Limonene	96	1029	1030	0.29	0.34	4.88	5.73
*β*-Phellandrene	94	1030	1031	0.07	0.08	1.15	1.31
Eucalyptol	97	1032	1032	0.62	0.75	11.42	13.82
(*Z*)-, β-Ocimene	90	1034	1035	0.01	0.01	0.16	0.16
(*E*)-, β-Ocimene	95	1045	1046	0.03	0.02	0.48	0.32
*γ*-Terpinene	95	1058	1058	15.18	6.01	24.29	9.62
3-Methylbut-2-enyl butanoate	90	1063	1068	0.06	0.06	1.00	1.00
(*Z*)-Sabinene hydrate	93	1070	1069	0.26	0.59	4.21	9.56
Terpinolene	96	1086	1086	0.09	0.08	1.62	1.44
*p*-Cymenene	94	1091	1093	0.01	0.02	0.17	0.35
Linalool	97	1099	1101	1.46	2.15	2.54	3.74
(*E*)-Sabinene hydrate	94	1102	1099	0.11	0.18	2.27	3.50
3-Methylbut-3-enyl 3-methylbutanoate	90	1110	1114	tr	0.02	0.00	0.36
(*Z*)-, *p*-Menth-2-en-1-ol	96	1126	1124	0.03	0.03	0.50	0.50
Camphor	97	1149	1149	0.27	0.36	5.40	7.20
Borneol	98	1173	1173	0.48	0.66	9.70	13.74
Terpinen-4-ol	92	1182	1184	0.66	0.63	12.32	11.76
Hex-(3Z)-enyl-Butyrate	92	1184	1187	0.01	0.03	0.18	0.54
*p*-Cymen-8-ol	93	1189	1189	0.02	0.05	0.40	1.00
*α*-Terpineol	97	1197	1195	0.12	0.15	2.26	2.82
(*Z*)-, Dihydro-carvone	94	1200	1198	0.03	0.05	0.56	0.93
*n*-Decanal	95	1206	1208	0.01	0.01	0.17	0.17
Thymol methyl ether	94	1230	1229	0.19	0.62	3.50	11.41
Carvacryl methyl ether	96	1239	1239	0.27	0.37	5.05	6.93
Neral	96	1242	1238	0.01	0.01	0.17	0.17
Carvone	95	1249	1246	0.01	0.02	0.18	0.36
Geranial	97	1274	1268	0.02	0.02	0.34	0.34
Thymol	94	1294	1293	55.81	54.21	1071.55	1040.83
Carvacrol	94	1302	1300	2.30	2.90	44.94	56.67
Thymol acetate	93	1345	1348	0.04	nd	0.80	0.00
Eugenol	95	1354	1357	0.05	0.12	1.06	2.54
Isobornyl propionate *	93	1376	1377	0.04	0.06	0.80	1.20
*α*-Copaene *	88	1377	1375				
*β*-Bourbonene	95	1385	1382	0.02	0.03	0.36	0.54
(*Z*)-Jasmone	93	1394	1394	nd	0.01	0.00	0.19
(*E*)-Caryophyllene	97	1421	1424	1.56	1.92	28.08	34.56
*β*-Copaene	94	1431	1433	0.02	0.03	0.38	0.56
*α*-Humulene	97	1457	1454	0.05	0.06	0.85	1.02
(*Z*)-Muurola-4(14),5-diene	94	1464	1466	tr	0.01	0.00	0.18
Geranyl propanoate	97	1468	1471	0.09	0.05	1.62	0.90
*γ*-Muurolene	92	1476	1478	0.05	0.06	0.90	1.08
*α*-Amorphene	90	1481	1482	nd	0.01	0.00	0.18
Germacrene D	95	1482	1480	0.11	0.04	1.87	0.68
*β*-Selinene	94	1491	1492	tr	0.01	0.00	0.18
*γ*-Amorphene	87	1494	1490	0.02	0.02	0.36	0.36
*α*-Selinene	89	1497	1501	0.01	0.01	0.18	0.18
*α*-Muurolene	93	1500	1497	0.03	0.03	0.53	0.53
*γ*-Cadinene	95	1515	1512	0.06	0.10	1.08	1.80
*δ*-Cadinene	94	1520	1518	0.11	0.11	1.98	1.98
(*E*)-Calamenene	90	1522	1527	0.02	0.03	0.40	0.60
(*E*)-Cadina-1,4-diene	93	1534	1536	0.01	0.01	0.18	0.18
*α*-Cadinene	95	1539	1538	0.01	0.01	0.18	0.18
Geranyl butyrate	97	1554	1559	0.02	0.02	0.36	0.36
Caryophyllene oxide	93	1585	1587	0.27	0.47	5.40	9.40
Humulene epoxide II	89	1613	1613	tr	0.01	0.00	0.19
(*Z*)-Cubenol	89	1618	1614	0.01	0.02	0.19	0.38
*epi*-γ-Eudesmol	95	1626	1624	0.05	0.03	0.90	0.54
α-Cadinol	94	1645	1641	0.06	0.12	1.29	2.58
Cadin-4-en-10-ol	95	1658	1659	0.04	0.02	0.73	0.37
Total				99.77	99.65		

Abbreviations: MS Sim, MS spectral similarity; LRI exp, experimental linear retention index; LRI ref, reference linear retention index; nd, not detected; tr, trace level; * indicates a coelution on SLB-5 ms column. ^a^ The volatile compounds are expressed in % values. ^b^ The relative concentrations were calculated based on the fact that the cells were treated with 200 μL essential oil emulsion.

## Data Availability

Data is contained within the article and supplementary material.

## Supplementary Materials

The following supporting information can be downloaded at: https://www.mdpi.com/article/10.3390/antiox11071330/s1, Figure S1: Measurement of reactive oxygen species after treatment of THP-1 cells with *P. aeruginosa* LPS.
