# Supplementary material for: Antioxidant and Anti-Inflammatory Effects of Thyme (Thymus vulgaris L.) Essential Oils Prepared at Different Plant Phenophases on Pseudomonas aeruginosa LPS-Activated THP-1 Macrophages
Source: Antioxidants (Basel). 2022 Jul 6;11(7):1330. doi: 10.3390/antiox11071330 (PMC9311800; doi:10.3390/antiox11071330)
Supplement: Supplementary file 1 [file antioxidants-11-01330-s001.zip › antioxidants-1794462-supplementary.pdf]

# Antioxidant and Anti-Inflammatory Effects of Thyme (*Thymus vulgaris L.)* Essential Oils Prepared at Different Plant Phenophases on *Pseudomonas aeruginosa* LPS Activated THP-1 Macrophages


**Edina Pandur [1], Giuseppe Micalizzi [2,3], Luigi Mondello [2,3,4], Adrienn Horváth [1], Katalin Sipos [1] and Györgyi Horváth [5,*]**

[1] Department of Pharmaceutical Biology, Faculty of Pharmacy, University of Pécs, H-7624, Rókus u. 2., Pécs, Hungary; katalin.sipos@aok.pte.hu, edina.pandur@aok.pte.hu, horvath.adrienn2@pte.hu

[2] Department of Chemical, Biological, Pharmaceutical and Environmental Sciences, University of Messina, 98168, Italy; giuseppe.micalizzi@chromaleont.it, lmondello@unime.it

[3] Chromaleont s.r.l., c/o Department of Chemical, Biological, Pharmaceutical and Environmental Sciences, University of Messina, 98168, Italy; giuseppe.micalizzi@chromaleont.it, lmondello@unime.it

[4] Unit of Food Science and Nutrition, Department of Medicine, University Campus Bio-Medico of Rome, 00128 Rome, Italy; lmondello@unime.it

[5] Department of Pharmacognosy, Faculty of Pharmacy, University of Pécs, H-7624, Rókus u. 2., Pécs, Hungary; horvath.gyorgyi@gytk.pte.hu

* Correspondence: horvath.gyorgyi@gytk.pte.hu


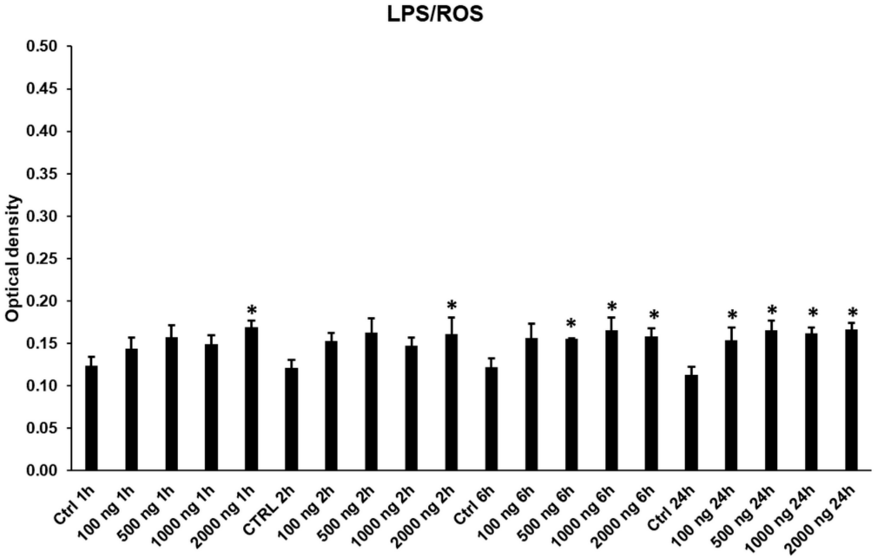


**Supplementary Figure S1.** Measurement of reactive oxygen species after treatment of THP-1 cells with *P. aeruginosa* LPS. THP-1 cells were treated with increasing concentration *P. aeruginosa* LPS (100 ng/mL - 2000 ng/mL) for different time periods (1 h – 24 h) to find the proper LPS concentration and incubation time for generating ROS. Asterisk marks $p < 0.05$ compared to the appropriate control (1 h, 2 h, 6 h and 24 h).
